# Effects of ankle–foot orthoses on gait parameters in post-stroke patients with different Brunnstrom stages of the lower limb: a single-center crossover trial

**DOI:** 10.1186/s40001-024-01835-2

**Published:** 2024-04-15

**Authors:** Fangchao Wu, Zhe Meng, Kezhen Yang, Jianhua Li

**Affiliations:** Department of Rehabilitation Medicine, Sir Run Run Shaw Hospital, Zhejiang University School of Medicine, No. 3, Qingchun East Road, Shangcheng District, Hangzhou, People’s Republic of China

**Keywords:** Ankle–foot orthoses, Stroke, Gait, Brunnstrom stage, Asymmetry

## Abstract

**Background:**

Ankle-foot orthoses (AFO) can improve gait posture and walking ability in post-stroke patients. However, the effect of AFO on gait parameters in post-stroke patients according to the Brunnstrom stage of stroke recovery of the lower limbs remains unclear. The study aimed to investigate whether stroke patients with different Brunnstrom stages benefit from wearing AFO.

**Methods:**

Twenty-five post-stroke participants included 18 men (50 ± 13 years) and 7 women (60 ± 15 years). The patients were divided based on Brunnstrom stage III or IV of the lower limbs. All patients underwent the gait and timed up and go (TUG) test using a gait analysis system while walking barefoot or with an AFO. The spatiotemporal and asymmetric parameters were analyzed.

**Results:**

All 25 patients completed the study. Significant differences were observed between barefoot and AFO use in TUG time (*P* < 0.001) but not walking velocity (*P* > 0.05). The main effect of the swing time ratio was significant in both groups (*P* < 0.05); however, the main effects of stride length, stance time, and gait asymmetry ratio were nonsignificant (*P* > 0.05). For barefoot versus AFO, the main effects of stride length (*P* < 0.05) and swing time (*P* < 0.01) ratios were significant, whereas those of stance time and gait asymmetry ratio were nonsignificant (*P* > 0.05).

**Conclusions:**

Post-stroke patients with lower Brunnstrom stages benefitted more from AFO, particularly in gait asymmetry.

**Supplementary Information:**

The online version contains supplementary material available at 10.1186/s40001-024-01835-2.

## Introduction

Stroke has become the leading cause of death and disability in China and the United States [1, 2]. Post-stroke patients often experience limb paralysis, and approximately 80% of survivors experience gait and postural abnormalities [3]. These abnormalities can decrease the patient's ability to perform activities of daily living, which can further affect their participation in work and social activities. Patients with post-stroke gait abnormalities exhibit decreased spatiotemporal and asymmetry parameters [4, 5]. Post-stroke patients often experience decreased gait function owing to foot drop accompanied by inversion [6]. Ankle–foot orthoses (AFO) are commonly used in rehabilitation training to improve gait parameters by promoting ankle dorsiflexion and toe clearance, suppressing excessive excitation of the triceps surae, and restoring walking function [7].

However, studies have found that several physical conditions significantly impact the gait parameters of post-stroke patients when applying AFO, including AFO type [8, 9], course of disease [10, 11], walking velocities [12], walking loads [13], adaptability designs [14], and walking slopes [15]. In addition, the Brunnstrom stage (BS) is a major influencing factor. The BS is the most commonly used staging scale for limb function recovery in post-stroke patients. Cho et al. found a significant association between gait level and BS of the lower limb [16]. However, it is unclear which BS (III or IV) benefits more from wearing an AFO, and the effects of wearing an AFO on gait parameters in patients with both stages have not been reported. AFOs are commonly used to train and improve gait function in post-stroke patients with lower limb BS of III and IV. Post-stroke patients with BS III and IV have some degree of lower limb mobility but do not develop adequate ankle dorsiflexion and toe contouring [17]. Previous studies have shown that wearing an AFO can improve ankle dorsiflexion and reduce foot drop. Patients with lower BS have relatively poorer lower limb separation movements and may have more severe ankle hyperextension or foot drop than those with higher BS. Therefore, patients with a lower BS may benefit from fitting an AFO as early as possible to improve gait abnormalities.

This study aimed to observe the effect of wearing an AFO on the gait of patients with different BS after stroke. It was hypothesized that stroke patients with different BS would experience different gait effects after wearing an AFO. Gait parameters, including timed up-and-go (TUG) test time, walking speed, step length ratio, stance time ratio, swing time ratio, and gait symmetry ratio, were assessed in patients with BS III and IV with and without AFO.

## Methods

### Participant-related information

The participants were 25 post-stroke patients with BS III and IV of the lower limbs (2 weeks and < 2 years after stroke onset, respectively) aged between 18 and 80 years. Patients in the subacute to chronic phase were selected for this study because their gait is significantly affected by AFO use in daily life. Participants were recruited from among inpatients or outpatients who could walk independently for > 10 m with or without assistive devices between November 2021 and November 2022 at the Rehabilitation Department of Sir Run Run Shaw Hospital. All participants had experienced a first-time ischemic stroke in the basal ganglia region with unilateral limb paralysis and had not used any kind of AFO in their daily lives in the previous 3 m. Patients with sensory impairment, communication problems, or cognitive problems were excluded. The exclusion criteria were severe lower limb spasticity that resulted in the inability to land on the affected heel, and Achilles tendon contracture. The study protocol was approved by the Ethics Committee of Sir Run Run Shaw Hospital, which is affiliated with Zhejiang University School of Medicine (approval no. 20201013-31). Written informed consent was obtained from all participants prior to their participation in the study.

### AFO used in this study

A nonarticulated AFO (50S1; Otto-Bock Co., Ltd., Duderstadt, Germany) was used in this study. Although this type of AFO is not the most advanced or suitable for every patient, it is the most easily purchased by patients in China and the most widely used by physiotherapists for gait training in post-stroke patients. It is a custom-made carbon-fiber AFO that allows no movement. This type of AFO inhibits ankle plantar flexion and inversion, and restricts ankle dorsiflexion. The AFOs used in this study are illustrated in Additional file 1: Fig. S1.

### Experimental protocol

This single-center, non-blinded, crossover trial included 25 participants: 18 men (50 ± 13 years) and 7 women (60 ± 15 years). All participants underwent walking tests at a self-selected gait speed on a 10 m trail in a randomized sequence by calibrated physiotherapists. All calibrated physiotherapists were master's degree holders with at least 5 years of experience.

Gait was measured using a 3D Gait Analysis System (QH-JBE-Y; Dalian Qianhan Technology Co., Ltd., Dalian, China) which has been subjected to clinical application research on gait analysis in China and has been confirmed to be reliable and effective. The gait analysis system consists of customized shoes of different sizes with built-in plantar pressure sensors placed at the bottom of the shoes to define gait cycles. The wearable sensing modules include six-axis inertial sensors placed behind the waist, thighs, calves, and feet that collect gait parameters during walking. Gait parameters include TUG test time, walking velocity, stride length ratio, stance time ratio, swing time ratio, and gait symmetry ratio. All sensor switches were turned on, and all patients completed the walking test in two states: wearing gait shoes with and without an AFO. The Gait Analysis System collected the gait parameters of all patients using a Bluetooth wireless transmission. After the gait test, all patients underwent another 3 m TUG [18] in a randomized sequence in the same two states, and time parameters were recorded. We defined participants wearing gait shoes without AFO as the barefoot (BF) group and those wearing gait shoes with AFO as the AFO group. Furthermore, we defined patients according to BS as the BS III and BS IV groups.

All gait parameters are presented as the average of at least three gait cycles for each condition, and patients were provided with sufficient rest time between tests. The researchers provided non-contact protection throughout the trial period to ensure patient safety. The use of a cane was permitted; however, it had to be used consistently when walking with or without an AFO and on the TUG [19]. All evaluations were performed by the same physiotherapist with 10 years’ experience.

### Data analysis and outcome measures

Gait parameters were collected and calculated using a computer software. Gait parameters included walking velocity and TUG test time as outcome measures.

To determine the presence and pattern of gait asymmetry, stride length, stance time, and swing time ratios were calculated, which are presented as the maximum divided by the minimum score, where 1 indicates perfect symmetry and larger scores represent Fgreater asymmetry on the left or right side [20, 21]. The gait asymmetry ratio was also calculated using the following equation [22]:

$$\mathrm{Gait \,symmetry \,ratio}=\frac{(\mathrm{paretic \,swing \,time}/\mathrm{paretic \,stance \,time})}{(\mathrm{nonparetic \,swing\, time}/\mathrm{nonparetic \,stance \,time})}$$

### Statistical analysis

Data were analyzed using SPSS (version 27.0; IBM, Armonk, NY, USA) at a significance level of 0.05. The Kolmogorov–Smirnov (K–S) test was used to test for normal distribution. Data with a normal distribution were analyzed using repeated-measures analysis of variance and simple effects analysis, with the BS and AFOs worn as variables. Variance values (*F*) and effect sizes (*η*^2^) were calculated. A simple effects analysis was performed to determine whether the interaction effect between BS and AFO was significant. If the main or simple effect differences were significant, post hoc analyses were performed using the Bonferroni correction to compare intra- or intergroup differences. Non-normally distributed data were analyzed using generalized estimation equations. If no interaction effect was noted between the two variables, only the main-effect analysis was considered.

## Results

### Demographic analysis of participants

All 25 patients completed the study. Age, weight, height, chronicity, sex, and the affected side did not differ significantly between the groups (*P* > 0.05; Table 1). Three of the eight participants in the BS III group and seven of the 17 in the BS IV group used a cane. Throughout the test, no unforeseen circumstances affected the results.

**Table 1:** Participants’ demographic characteristics

	Age, years	Weight, kg	Height, cm	Chronicity, months	Sex		Affected side	
					Male	Female	Left	Right
BS III group	50.25 (15.25)	68.06 (10.13)	165.75 (4.37)	5.13 (4.88)	7	1	4	4
BS IV group	58.35 (12.58)	64.74 (9.17)	164.29 (5.50)	7.12 (11.22)	11	6	7	10

Data are presented as the mean (standard deviation)

*BS III* Brunnstrom stage III; *BS IV* Brunnstrom stage IV

### Spatiotemporal parameters analysis

**Fig. 1:**
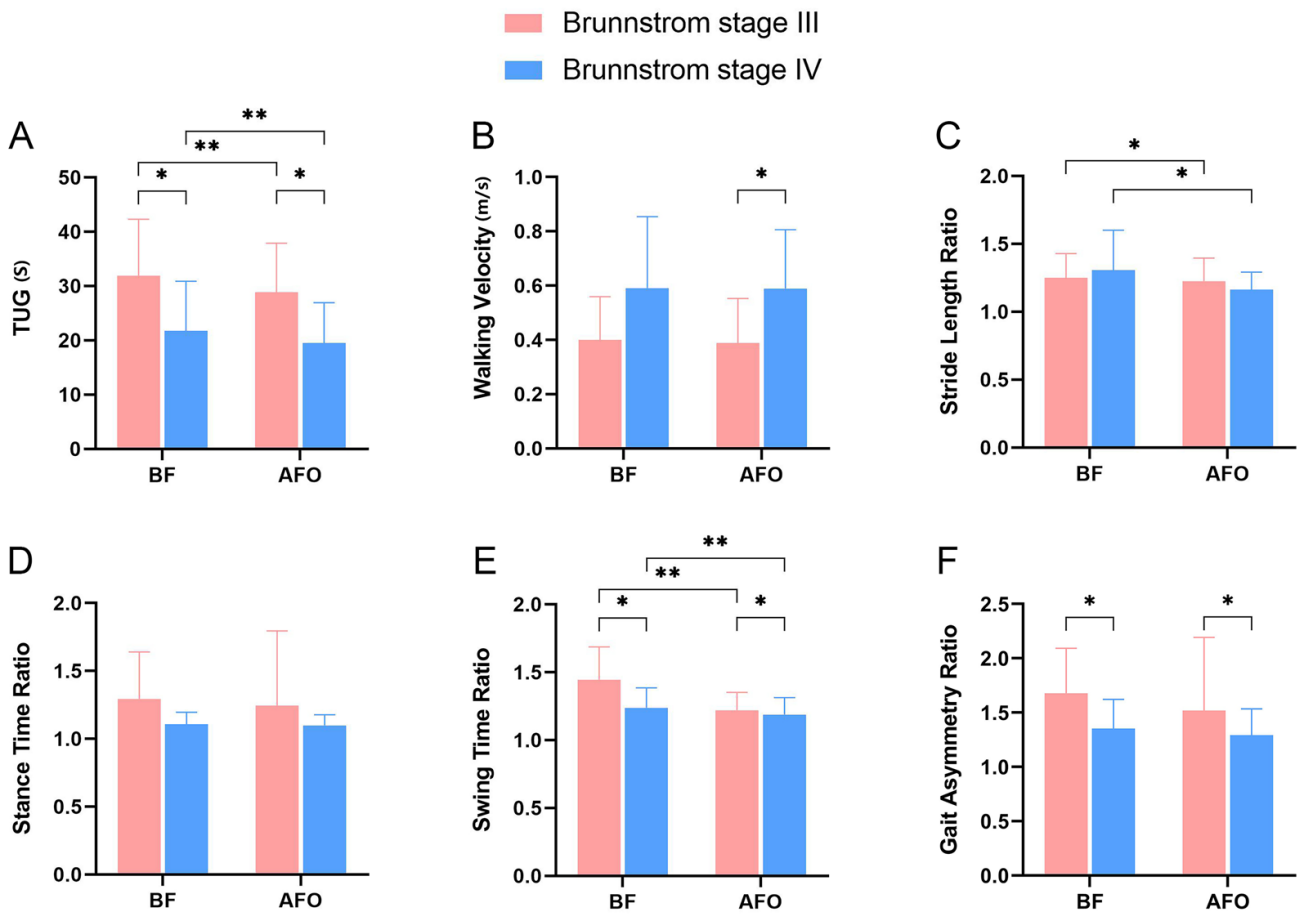
Comparisons of **A** TUG time, **B** walking velocity, **C** stride length ratio, **D** stance time ratio, **E** swing time ratio, and **F** gait symmetry ratio. The *X*-axis represents the different groups. AFO group, wearing gait shoes with AFO; BF group, wearing gait shoes without AFO. AFO, ankle–foot orthoses; BF, barefoot; TUG, timed up-and-go test

After the *K*–*S* test, the TUG and walking velocity data were normally distributed. The spatiotemporal parameter analysis results are shown in Fig. 1A–B, and the results of the post hoc analysis are shown in Table 2. The TUG showed a significant main effect of BS III versus IV (*F* = 6.950, *P* = 0.015, *η*^2^ = 0.232) and BF versus AFO (*F* = 16.363, *P* < 0.001, *η*^2^ = 0.416) and a nonsignificant interaction effect of the two variables (*F* = 0.414, *P* = 0.527, *η*^2^ = 0.018). Post hoc comparisons revealed significant differences between BS III and IV in BF (*P* < 0.05) and AFO (*P* < 0.05). In patients with BS III and BS IV, the difference between BF and AFO was significant (both *P* < 0.01). For walking velocity, the main effect was significant for BS III versus IV (*F* = 0.419, *P* = 0.045, *η*^2^ = 0.164) and nonsignificant for BF versus AFO (*F* = 0.107, *P* = 0.747, *η*^2^ = 0.005), and the interaction effect between the two variables was nonsignificant (*F* = 0.070, *P* = 0.794, *η*^2^ = 0.003). Post hoc comparisons revealed that the difference between BS III and IV was not significant for BF (*P* > 0.05) but was significant for AFO (*P* < 0.05). No significant difference was observed between BF and AFO for either BS III or IV (*P* > 0.05). For post-stroke participants, a higher TUG test score and higher walking velocity were beneficial for walking ability. These results suggest that wearing an AFO is more helpful in improving the TUG and walking speed in patients with BS IV.

**Table 2:** Post hoc analysis of spatiotemporal parameters

	TUG (s)			Walking velocity (m/s)		
	BS III	BS IV	*P/F*/*η*^2^	BS III	BS IV	*P/F*/*η*^2^
e	31.93 ± 10.37	21.74 ± 9.16	*P* < 0.05	0.40 ± 0.16	0.59 ± 0.26	*P* > 0.05
			*F* = 6.202			*F* = 3.550
			*η*^2^ = 0.212			*η*^2^ = 0.134
e	28.89 ± 9.01	19.53 ± 7.42	*P* < 0.05	0.39 ± 0.16	0.59 ± 0.22	*P* < 0.05
			*F* = 7.555			*F* = 5.393
			*η*^2^ = 0.247			*η*^2^ = 0.190
*P/F*/*η*^2^	*P* < 0.01	*P* < 0.01		*P* > 0.05	*e*	
	*F* = 8.081	*F* = 9.042		*F* = 0.128	*F* = 0.003	
	*η*^2^ = 0.260	*η*^2^ = 0.282		*η*^2^ = 0.006	*η*^2^ < 0.001	

Data are expressed as mean ± standard deviation

*AFO* ankle–foot orthoses, *BF* barefoot, *BS* Brunnstrom stage, *TUG* Timed Up and Go test

### Asymmetry parameter analysis

The *K*–*S* test showed that the stride length, stance time, swing time, and gait asymmetry ratio data were not normally distributed. The asymmetric parameter analysis results are shown in Fig. 1C–F, and the main effect analysis results are presented in Table 3. For stride length ratio, the main effect of BS III versus IV was not significant (*P* > 0.05), whereas that of BF versus AFO was significant (*P* < 0.05), and the interaction effect was nonsignificant (χ = 1.590, *P* > 0.05). For stance time ratio, the main effect of BS III versus IV was nonsignificant (*P* > 0.05), that of BF versus AFO was nonsignificant (*P* > 0.05), and the interaction effect of the two variables was nonsignificant (χ = 0.028, *P* > 0.05). For swing time ratio, a significant main effect was noted for BS III versus IV (*P* < 0.05) and BF versus AFO (*P* < 0.01) with a nonsignificant interaction effect (χ = 3.347, *P* > 0.05). For gait asymmetry ratio, the main effect of BS III versus IV was significant (*P* < 0.05), the main effect of BF versus AFO was nonsignificant (*P* > 0.05), and the interaction effect was nonsignificant (χ = 0.111, *P* > 0.05). For post-stroke participants, higher stride length ratios, standing time ratios, swing time ratios, and gait asymmetry ratios were more beneficial to walking ability. These results suggest that wearing an AFO can significantly improve the stride length, swing time, and gait asymmetry ratios in patients with BS III or IV; however, the stance time ratio cannot be significantly improved. Patients with BS III showed greater improvements when wearing the AFO.

**Table 3:** Main effects analysis of asymmetric parameters

	Stride length ratio		Stance time ratio		Swing time ratio		Gait asymmetry ratio	
	BS III	BS IV	BS III	BS IV	BS III	BS IV	BS III	BS IV
BF	1.25 ± 0.18	1.31 ± 0.29	1.29 ± 0.35	1.11 ± 0.09	1.68 ± 0.41	1.35 ± 0.27	1.45 ± 0.24	1.24 ± 0.15
AFO	1.23 ± 0.17	1.16 ± 0.13	1.25 ± 0.55	1.10 ± 0.08	1.52 ± 0.67	1.29 ± 0.24	1.22 ± 0.13	1.19 ± 0.12
BS III vs. IV	*P* > 0.05, χ = 0.002		*P* > 0.05, χ = 3.118		*P* < 0.05, χ = 4.787		*P* < 0.05, χ = 4.856	
BF vs. AFO	*P* < 0.05, χ = 5.351		*P* > 0.05, χ = 0.073		*P* < 0.01, χ = 8.464		*P* > 0.05, χ = 0.894	

*AFO* ankle–foot orthoses, *BF* barefoot, *BS* Brunnstrom stage

## Discussion

This study aimed to observe the influence of wearing an AFO on gait parameters among post-stroke patients and to provide clinical practice evidence for the application of an AFO in patients with different BS. Consistent with the initial hypothesis, stroke patients at different BS stages showed different levels of gait improvement after wearing an AFO. Post-stroke patients with a higher BS had better gait parameters and walking function. Moreover, the improvement in the gait parameters after wearing the AFO was significant. In contrast, post-stroke patients with a lower BS may have poorer gait parameters; however, wearing an AFO can yield greater improvements in gait parameters.

The 3-m TUG test was used to assess walking and motor function [23]. It is crucial to acknowledge that various physical conditions affect walking function, with different body structures or the decision to wear an AFO being significant influencing factors [24, 25]. The study found that the TUG time improved in both patient groups during AFO walking compared with BF walking. Additionally, the higher the BS, the better the TUG test time performance in post-stroke patients, which is consistent with previous studies [26, 27]. Akay [28] suggested that the fractal dimensions of body motion in post-stroke patients are closely related to the BS. AFOs provide support for walking stability during sitting, standing, and turning movements [29].

A significant improvement was found in walking velocity in patients with different BS. This is consistent with a previous study that showed an increase in walking velocity with an improvement in BS [30]. These results suggest that post-stroke patients with a higher BS exhibit better gait parameters. The use of an AFO can improve gait function by increasing walking velocity. No significant increase was found in walking speed when ankle-foot orthoses were worn, indicating that adaptability factors for wearing AFO must be considered. Stroke patients often walk in abnormal patterns for extended periods, making it difficult to adjust to changes in plantar sensation after wearing an AFO. This can result in inconsistent improvements in gait parameters. The self-selected gait speed used in this study may have influenced the subjective changes in gait parameters.

Gait asymmetry parameters indicate the degree of asymmetry in a patient’s bilateral lower limbs during walking [31]. The gait asymmetry ratio in this study was close to 1, indicating better symmetry in both lower limbs, which was related to improved gait function. Research has shown that the application of an AFO can effectively improve the gait asymmetry ratio [32].

Based on these findings, changes occurred in several gait asymmetry parameters after wearing AFOs. In patients with BS III, there was a significant difference in the stride length ratio and swing time ratio before and after wearing the AFOs. Although some indicators did not show statistical significance, improvement was greater in patients with BS III than in those with BS IV. Among them, gait asymmetry, a valid indicator of gait symmetry, improved more in the BS III group than in the BS IV group after wearing the AFO. These results are partially consistent with those of Nolan et al., which indicated that AFO walking can significantly improve stance time and stride length asymmetry [33]. It is important to consider foot proprioception as a factor that differs from previous research. This study identified differences in gait parameters between the current study and previous studies owing to the relatively short period of time that the AFOs were worn and the patients' lack of adaptation to wearing them. Additionally, the impact of AFOs on gait parameters is influenced by various factors such as inclusion criteria, disease course, motor function, number of cases, cooperation, and type of AFO [34]. The use of an AFO effectively improves toe clearance and ankle dorsiflexion, making it particularly beneficial for patients with BS III when walking. The study hypothesis was partially supported by the study results, as a decrease was observed in the difference in gait parameters between the two patient groups after wearing the AFO. However, further studies with larger sample sizes are needed to confirm this hypothesis.

This study had several limitations. The study did not provide additional details regarding the type of stroke experienced by the participants. It is important to note that strokes triggered by different factors may have varying effects on a patient's motor function. The limited number of participants who met the inclusion criteria and completed the trial, as well as the small number of female participants, may have affected the results. This study observed immediate changes resulting from AFO use; however, no observations of gait parameters were made after several weeks of training. Additionally, the use of a cane may be a contributing factor affecting the results. The effects of different AFO types on patient gait parameters were beyond the scope of this study. However, it should be noted that all types of AFOs affect gait parameters. Therefore, future studies should investigate the effects of different AFO types in patients with varying BS. Further well-designed randomized controlled clinical trials are needed to establish better scientific evidence for the effects of AFO use on gait variables in post-stroke patients [35].

## Conclusion

In conclusion, post-stroke patients with a higher BS have better walking function and gait parameters. Post-stroke patients with a lower BS tended to benefit more from AFO use in terms of gait parameters and asymmetry. Therefore, AFO should be used early in post-stroke patients to improve walking function and gait parameters. Our findings provide insights into the improvements in gait function and asymmetry during walking with AFO at different BS grades.

### Supplementary Information

**Additional file 1: Figure S1.** AFO used in this study.

## Data Availability

Data supporting the findings of this study are available upon request from the corresponding authors.

## Abbreviations

AFO: Ankle-foot orthosis
BF: Barefoot
BS: Brunnstrom stage
K–S: Kolmogorov–Smirnov
TUG: Timed up-and-go
