# Supplementary figures and images for: Effects of ankle–foot orthoses on gait parameters in post-stroke patients with different Brunnstrom stages of the lower limb: a single-center crossover trial
Source: Eur J Med Res. 2024 Apr 15;29:235. doi: 10.1186/s40001-024-01835-2 (PMC11017542; doi:10.1186/s40001-024-01835-2)

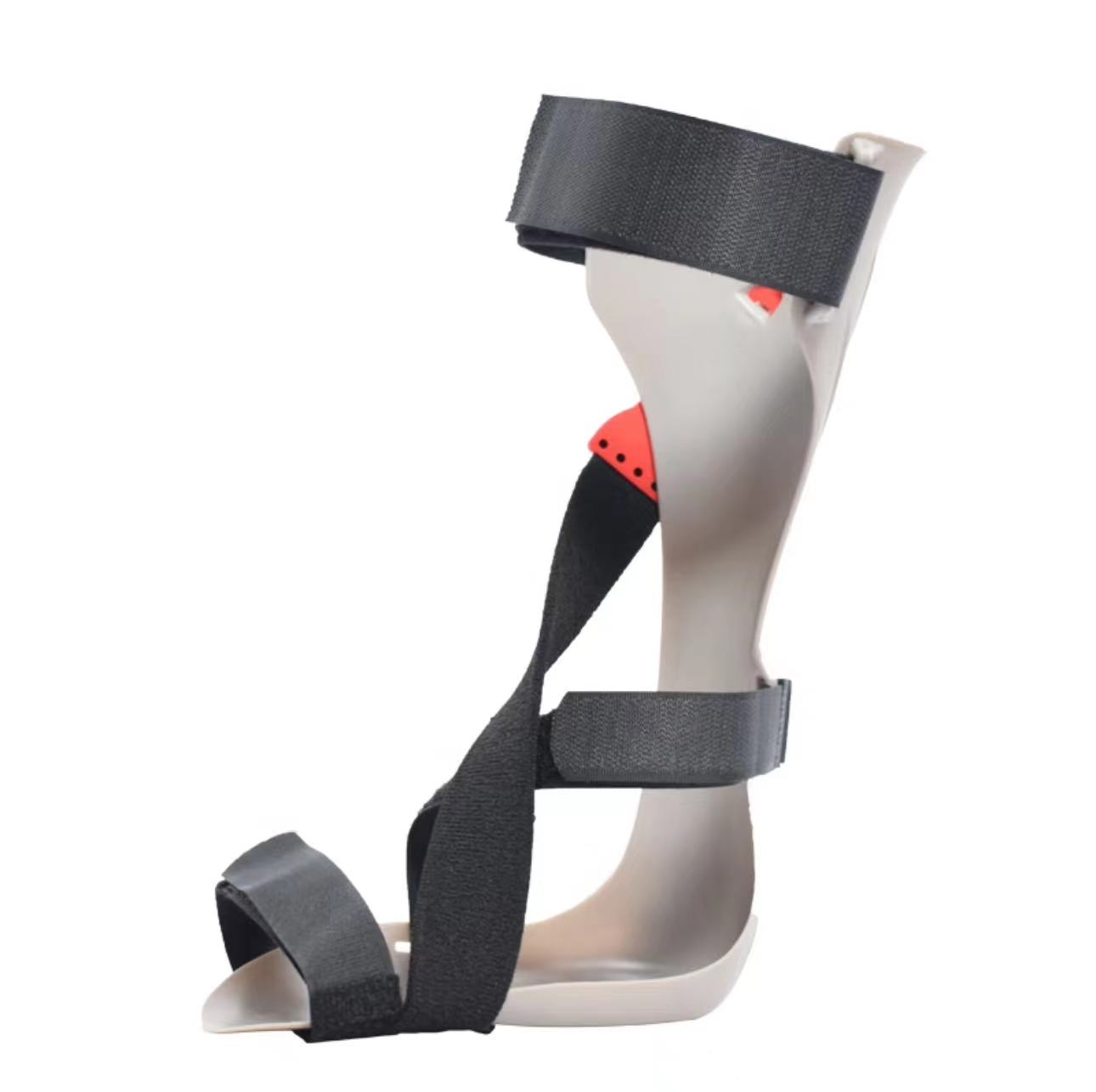


**Additional file 1: Figure S1.** AFO used in this study.

Supplement: Supplementary file 1 — Additional file 1: Figure S1. AFO used in this study. [file 40001_2024_1835_MOESM1_ESM.docx]
